# TNKPVI, a Putative Bioaccessible Pharmacophore of Anti-Inflammatory Potato Patatin-Derived Decapeptide DIKTNKPVIF

**DOI:** 10.3390/molecules27123869

**Published:** 2022-06-16

**Authors:** Emeka B. Okeke, Raliat O. Abioye, Esmeiry Ventura-Santana, Xiaohong Sun, Chibuike C. Udenigwe

**Affiliations:** 1 Department of Biology, College of Liberal Arts and Sciences, The State University of New York at Fredonia, Fredonia, NY 14063, USA; vent9552@fredonia.edu; 2 Department of Chemistry and Biomolecular Sciences, Faculty of Science, University of Ottawa, Ottawa, ON K1N 6N5, Canada; rabio069@uottawa.ca; 3 School of Nutrition Sciences, Faculty of Health Sciences, University of Ottawa, Ottawa, ON K1H 8M5, Canada; xsun5@uottawa.ca

**Keywords:** bioactive peptides, patatin, biostability, digestive proteases, bioaccessibility, bioinformatics, anti-inflammatory property, monocytes, cytokines, nutraceuticals

## Abstract

Potato protein-derived decapeptide DIKTNKPVIF exerted anti-inflammatory activity in animal models when delivered via intragastric gavage and intraperitoneal injection. However, DIKTNKPVIF is susceptible to hydrolysis in the digestive tract, which will decrease its bioaccessibility and possibly bioactivity. In this study, the anti-inflammatory activity of fragments generated from in silico gastrointestinal enzymatic hydrolysis of DIKTNKPVIF was investigated using the human monocytic (THP-1) cell line. The simulated digestion by pepsin and trypsin released four fragments, DIKTNKPVI, TNKPVIF, DIK and TNKPVI. The peptides lacked the cleavage sites of chymotrypsin. All five peptides were predicted to be non-toxic, which was validated using cytotoxicity assay at 0.25–1 mM peptide concentration. However, the peptides were predicted to possess poor pharmacokinetic profiles, including low passive gastrointestinal absorption and blood–brain barrier permeability. TNKPVIF, DIK and TNKPVI significantly reduced the amount of pro-inflammatory interleukin (IL)-6, IL-8 and tumor necrosis factor in lipopolysaccharide-activated THP-1 cells. Notably, the anti-inflammatory activity of fragment TNKPVI was comparable to that of the parent decapeptide while peptide fragment DIKTNKPVI had no apparent effect on the pro-inflammatory cytokines. This highlights the important role of the C-terminal phenylalanine residue of the parent peptide in the bioactivity. Furthermore, given its activity and the absence of cleavage sites of major digestive proteases, TNKPVI could be the biostable and bioaccessible pharmacophore of potato patatin-derived anti-inflammatory decapeptide DIKTNKPVIF.

## 1. Introduction

Food-derived peptides have shown a wide range of health-promoting bioactivities [1,2]. However, the importance or practical uses of these peptides largely depend on their biostability, which is seldom taken into consideration in the past. Bioactive peptides delivered orally encounter several proteases, including pepsin, trypsin, chymotrypsin and brush border peptidases, which may hydrolyze and deactivate them irreversibly during transit through the gastrointestinal tract. To circumvent this issue, different delivery systems, such as biopolymer-based nanoparticles, have been explored for the protection of bioactive peptides [3] in order to escape the gastrointestinal digestive proteases. Despite the strong prospects, this approach is still at the development stage as it can complicate the food matrix composition, reduce biocompatibility and increase the cost of production of the nutraceutical or functional food product [3,4]. Indeed, the absence of recognition and cleavage sites of gastrointestinal proteases on bioactive peptides reduces their susceptibility to hydrolysis during oral delivery. However, the potential for miscleavage and nonspecific cleavage by proteases has led to the recent proposal of additional structural requirements for biostability of food-derived bioactive peptides [5]. Therefore, it is crucial to integrate the structural biostability factors during the selection and rational design of orally administered food-derived bioactive peptides in order to increase their bioaccessibility.

Several food-derived peptides have demonstrated anti-inflammatory activities in vitro and in vivo, mostly through the inhibition of components of the inflammation signaling pathways [6]. For instance, the daily administration of DIKTNKPVIF, a decapeptide from Alcalase-hydrolyzed potato protein, to spontaneously hypertensive rats (SHR) and senescence-prone mice (SAMP8 mice) for eight weeks attenuated pro-inflammatory reactions via the MAPK pathway. The peptide decreased the protein expression of pro-inflammatory Toll-like receptor 4, phosphor-nuclear factor κB, phosphor-p38 MAPK, tumor necrosis factor (TNF) and interleukin (IL)-6 during hypertension-induced cardiac inflammation in SHR and high-fat diet-induced inflammation in SAMP8 mice [7,8]. However, the peptide was administered to the animals via intragastric gavage and intraperitoneal injection, which pose some challenges for food and nutraceutical applications. In fact, both studies concluded that the intact form of DIKTNKPVIF exhibited anti-inflammatory activity in vivo; however, the peptide contains the cleavage sites of major gastrointestinal proteases. Hydrolysis of bioactive peptides by gastrointestinal proteases can lead to loss or gain of bioactivity, or no effect. For example, in vitro digestion of decapeptide AKSLSDRFSY by pancreatic proteases resulted in the release of peptide fragments LSDRFS, SDRFSY, SLSDRFS and SLSDRFSY, with either the loss or retention of antihypertensive property of the parent peptide in cultured vascular smooth muscle cells [9]. Other examples include octapeptide EDEVSFSP from soybean protein isolate fermented by *Pediococcus pentosaceus* SDL1409 and heptapeptide YPFPGPI from bovine milk casein, both of which exhibited increased angiotensin-converting enzyme and dipeptidyl peptidase-IV inhibitory activities, respectively, following hydrolysis by gastrointestinal proteases [10,11].

In order to advance the development of the potato-derived DIKTNKPVIF for nutraceutical application, it is important to understand its biostability–bioactivity relationship. In this study, in silico tools were used to simulate enzymatic hydrolysis of DIKTNKPVIF in the gastric and intestinal phases of digestion, and anti-inflammatory activity of the parent peptide and resulting fragments was evaluated using the human monocytic (THP-1) cell line. The objectives of this study were to evaluate the in silico gastrointestinal-released peptide fragments for loss, gain or retention of anti-inflammatory activity compared to DIKTNKPVIF, predict their pharmacokinetic profiles and identify the biostable and bioaccessible anti-inflammatory pharmacophore of the potato peptide.

## 2. Materials and Methods

### 2.1. In Silico Gastrointestinal Hydrolysis

Biostability of peptide DIKTNKPVIF was determined using ExPASy PeptideCutter (https://web.expasy.org/peptide_cutter/ (accessed on 7 June 2021)), which predicts potential cleavage sites of proteases. Major gastrointestinal proteases selected for this analysis include pepsin (pH1.3) for the gastric phase, and chymotrypsin high specificity (C-term to [FYW], not before P), chymotrypsin low specificity (C-term to [FYWML], not before P) and trypsin for the intestinal phase. The parent peptide and fragments generated were further analyzed for physicochemical and pharmacokinetic properties in silico and anti-inflammatory activity in vitro.

### 2.2. Physicochemical Properties, Pharmacokinetics and Target Predictions

UniProt BLAST (Basic Local Alignment Search Tool; https://www.uniprot.org/blast/; accessed on 22 March 2022) was used for the similarity search of Swiss-Prot and TrEMBL to identify the parent potato protein and location of DIKTNKPVIF. Physicochemical properties of the parent peptide and resulting fragments were calculated using the ‘Peptides’ package in *R* (https://github.com/dosorio/Peptides/; accessed on 1 February 2022). SMILES strings of the peptides were obtained from BIOPEP-UWM. Pharmacokinetics and drug-likeness of the peptides were determined using SwissADME (http://www.swissadme.ch/index; accessed on 7 June 2021), which predicts the absorption, distribution, metabolism and excretion properties [12]. Potential toxicity of the parent peptide and fragments was predicted using ToxinPred (https://webs.iiitd.edu.in/raghava/toxinpred/index.html; accessed on 7 June 2021) [13]. For some fragments, estimation of the most probable macromolecular target(s) for inflammatory activity was carried out using SwissTargetPrediction (http://www.swisstargetprediction.ch/; accessed on 19 March 2022), with *Homo sapiens* selected as the target species [14].

### 2.3. Peptide Synthesis

The parent peptide and four fragments were synthesized and supplied as white powders by Bootech BioScience and Technology Co., Ltd. (Shanghai, China). Purity was determined by the supplier to be >95% after observing the peptide peak by analytical high-performance liquid chromatography and mass spectrometry analysis. The sequence and experimental mass of the peptides are provided in Table 1.

### 2.4. Chemicals and Cell Culture

The human monocytic leukemia cell line THP-1 (American Type Culture Collection—ATCC, Manassas, VA, USA) was grown in RPMI 1640 culture medium (ATCC) supplemented with 10% fetal bovine serum (FBS) and 1% penicillin/streptomycin both from Thermo Fisher Scientific (Waltham, MA, USA). Cells were cultured at 37 °C in 5% CO_2_ in a humidified incubator. Media were renewed every 3 days and cells were sub-cultured when the concentration reached 8 × 10^5^ cells/mL. Cells were discarded and replaced by frozen stocks after 20 passages. Cytotoxicity kit for determining cell viability based on lactate dehydrogenase (LDH) release was purchased from Thermo Fisher Scientific. Lipopolysaccharide (LPS) was purchased from Millipore-Sigma (St. Louis, MO, USA). ELISA kits were purchased from Thermo Fisher Scientific (Waltham, MA, USA).

### 2.5. Human Monocyte-Derived Macrophages and Cytotoxicity Assay

Human monocyte (THP-1) cells were differentiated into macrophages in 96-well plates using phorbol 12-myristate 13-acetate (PMA; Millipore Sigma, St. Louis, MO, USA) as previously reported [15]. PMA was removed and the adherent macrophages were washed and rested for 24 h. Varying concentrations of the test peptides were added to the cell culture for 24 h and culture supernatants were used to determine the cytotoxicity of peptides according to the manufacturer’s instructions. Cell viability was obtained based on LDH release by comparing data obtained from peptide-treated macrophages to those of untreated cells.

### 2.6. Cytokine Assay

Human monocyte (THP-1) cells were differentiated into macrophages in 96-well plates using PMA. PMA was removed and the adherent macrophages were washed and rested for 24 h. Cells were treated with peptides for 1 h followed by activation with LPS (1 µg/mL) for 12 h. The levels of IL-6, IL-8 and TNF in culture supernatants were determined using ELISA kits according to the manufacturer’s instructions.

### 2.7. Statistical Analysis

Cell culture experiments were conducted in triplicate, and cytotoxicity and cytokine data expressed as mean values ± standard deviation (S.D.). A one-tailed Student *t* test was performed using GraphPad Prism version 9.2.0 for Windows (GraphPad Software, La Jolla, CA, USA) to compare the differences in cytokine production between LPS and peptide-treated groups.

## 3. Results and Discussion

### 3.1. In Silico Gastrointestinal Biostability and Physicochemical Properties

**Figure 1:**
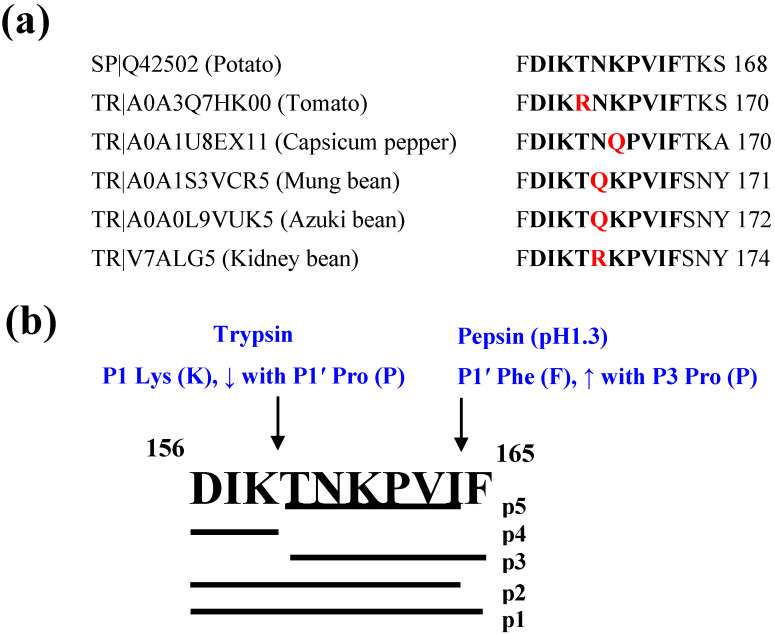
(**a**) Sequence alignment and location of DIKTNKPVIF or its variants on patatin sequences of potato and other plant-based foods retrieved from Swiss-Prot (SP) or TrEMBL (TR). Red letters indicate sequence variation relative to the potato patatin peptide sequence (**b**) Cleavage sites of two major gastrointestinal proteases on DIKTNKPVIF (f156–165; p1) resulting in the release of peptide fragments DIKTNKPVI (f156–164; p2), TNKPVIF (f159–165; p3), DIK (f156–158; p4) and TNKPVI (f159–164; p5).

The similarity search returned 34 entries with 100% identity in Swiss-Prot for the potato-derived anti-inflammatory decapeptide, DIKTNKPVIF, all originating from *Solanum tuberosum* patatin (f156–165). As shown in Figure 1a, TrEMBL search showed different variants of the peptide, also found in patatin, from other plant-based foods such as tomato, capsicum pepper and various legumes. Patatin, a glycoprotein, is the major storage protein of potato and constitutes over 40% of the soluble proteins in the tuber [16]. In a previous study, DIKTNKPVIF was released from a 2.5% crude potato protein using Alcalase [8]. Peptidomic analysis showed that the decapeptide was also released from a potato protein isolate after treatments with pepsin from porcine gastric mucosa (peptide 29) or a combination of pepsin and pancreatin from porcine pancreas (peptide 61) [17]. Although the yields were not provided, findings from these studies indicate that the anti-inflammatory decapeptide can be sustainably produced for nutraceutical application, given the wide availability of the starting protein material and releasing enzymes.

DIKTNKPVIF is susceptible to hydrolysis by proteases in the digestive tract, which would affect its biostability. As shown in Figure 1b, in silico digestion of the decapeptide (p1) using pepsin (pH 1.3) and trypsin, two major proteolytic enzymes of the gastrointestinal tract, resulted in the production of four fragments, DIKTNKPVI (p2), TNKPVIF (p3), DIK (p4) and TNKPVI (p5). Cleavage of DIKTNKPVIF at the C-terminal Phe at P1′ by pepsin resulted in the release of DIKTNKPVI, and further hydrolysis by trypsin at P1 Lys resulted in the formation of DIK and TNKPVI fragments. In addition, cleavage of the parent peptide by trypsin at P1 Lys also liberated DIK to create TNKPVIF, which is equally susceptible to additional cleavage at P1′ Phe by pepsin. Similar to the parent peptide, fragments DIKTNKPVI and TNKPVIF are not biostable as they are susceptible to further hydrolysis by pepsin or trypsin. The hydrolytic effect of pepsin at P1′ Phe is enhanced when Pro is present at the P3 location [18], as found in DIKTNKPVIF and TNKPVIF (Figure 1b). However, fragments DIK and TNKPVI are resistant to further degradation by the proteases, indicating potential gastrointestinal biostability. Notably, the Lys of TNKPVI in P1 position was not hydrolyzed by trypsin due to the blocking effect of the P1′ Pro of the hexapeptide (Figure 1b) [18]. C-terminal Phe was recently reported to reduce biostability of bioactive peptides [5] as it is a major cleavage site of pepsin especially in the absence of Arg, Lys or His at P3 position and Arg at P1 position [18]. Both the low- and high-specificity chymotrypsin did not hydrolyze the parent peptide or fragments as the protease only cleaves at Phe in the P1 position.

The structural characteristics of the parent peptide and resulting fragments are presented in Table 1. Generally, fragments with larger molecular weights were less theoretically biostable compared to smaller peptides, possibly because longer sequences have a higher chance of possessing more cleavage sites of the gastrointestinal proteases compared to shorter sequences [5,19]. In addition, low hydrophobicity, high negative charge at pH 7.0 and a high number of acidic residues have been associated with increased peptide biostability [20]. However, these structural properties are largely absent in the patatin peptide and digestive fragments. Peptide binding potential to membrane and other proteins is represented by the Boman index, where values above 2.48 indicate high protein binding capabilities [21]. DIK was the only peptide with high protein binding potential and it was also the most stable based on the instability index (Table 2). In general, an instability index value below 40 indicates that the peptide is predicted to be stable [22]. Consequently, TNKPVI was predicted to be unstable. Furthermore, the aliphatic index values of DIKTNKPVIF and its fragments (Table 2) indicate that the peptides have a high degree of thermostability over a wide range of temperatures [23].

### 3.2. Pharmacokinetic Properties of the Parent Peptide and Fragments

To evaluate the pharmacokinetic properties and potential toxicity of the peptide fragments, in silico ADME/Tox analysis was performed (Table 2). All the peptides and fragments were predicted to be non-toxic based on the absence of amino acid/dipeptide composition or motifs abundant in toxic peptides [13]. However, the peptides have low passive gastrointestinal absorption and blood–brain barrier permeability (Table 2). Interestingly, the peptides lack the ability to inhibit cytochrome P450 3A4 and only TNKPVI was predicted to not be a substrate to P-glycoprotein 1. P-glycoprotein 1 or multidrug resistance protein 1 is an efflux transporter responsible for pumping a wide range of xenobiotic compounds out of cells, including enterocytes, thus regulating their bioaccumulation [24]. Consequently, TNKPVI is more likely to resist the efflux activity of P-glycoprotein 1 leading to its retention and accumulation in cells compared to the parent peptide and other fragments. This can increase the cytosolic concentration and bioavailability of the peptide. However, all the peptides except DIK had low bioavailability scores; DIK had a bioavailability score of 0.55, indicating that it passed the rule-of-five and thus showed a higher probability of >10% bioavailability or measurable Caco-2 permeability [25,26]. High molecular flexibility and polarity resulted in rotatable bonds (ROTB) greater than 10 and topological polar surface area (TPSA) greater than 140 for all the peptide fragments, which indicate low predicted oral bioavailability [27]. Lastly, DIKTNKPVI, DIK and TNKPVI were predicted to be highly soluble in water and the parent peptide, DIKTNKPVIF and fragment TNKPVIF were very soluble (Table 2) [28].

### 3.3. Anti-Inflammatory Activity of the Parent Peptide and Fragments

**Figure 2:**
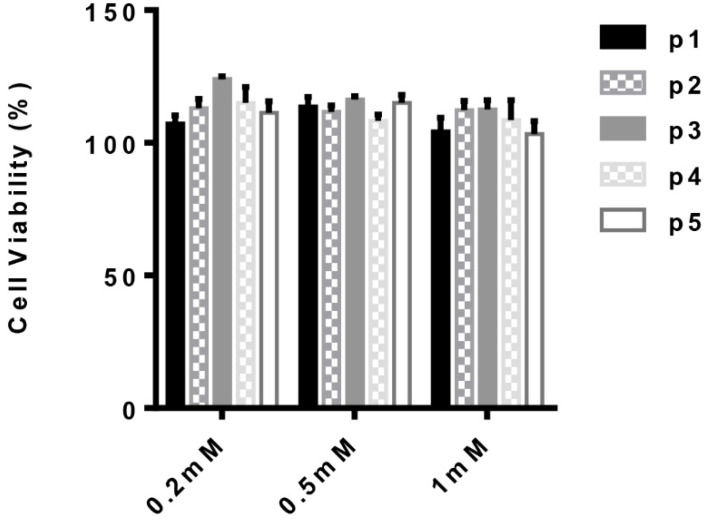
Cell viability of human monocyte (THP-1) cells treated with DIKTNKPVIF (p1) and its digestive fragments DIKTNKPVI (p2), TNKPVIF (p3), DIK (p4) and TNKPVI (p5) compared to untreated cells. Bars represent mean values ± S.D.

The anti-inflammatory activity of the parent peptide, DIKTNKPVIF (p1), has been previously reported [7,8]. The present study sought to determine if any of the newly obtained peptide fragments retained the anti-inflammatory activity of the parent peptide by investigating their ability to suppress LPS-induced inflammatory responses in cultured human monocytes. Initially, the safety of the peptides was established based on the membrane integrity of human monocyte-derived macrophages and LDH release into the culture media in compromised cells. As seen in Figure 2, neither the parent peptide nor the digestive fragments showed any cytotoxic effect up to a peptide concentration of 1 mM. This finding confirms the lack of toxicity predicted by ToxinPred (Table 2) and indicates that the peptides are potentially safe for use as active principles in the development of nutraceuticals for human consumption.

**Figure 3:**
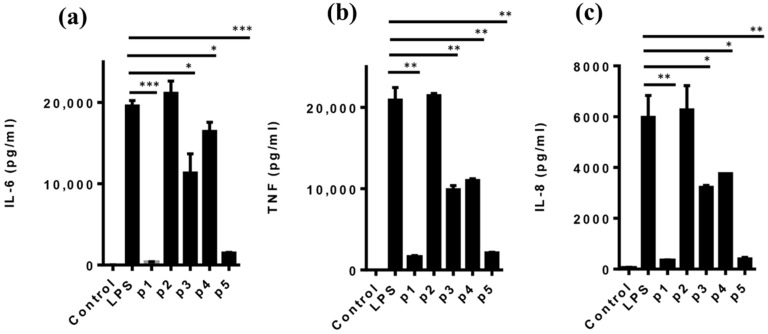
Effect of peptides on LPS-activated monocyte-derived macrophages. Cells were treated with 1 mM of each of the peptides DIKTNKPVIF (p1), DIKTNKPVI (p2), TNKPVIF (p3), DIK (p4) and TNKPVI (p5) for 1 h before activation with LPS to induce inflammation. The levels of pro-inflammatory cytokines (**a**) interleukin (IL)-6, (**b**) tumor necrosis factor (TNF) and (**c**) IL-8 in the culture supernatant were determined by ELISA. Values are mean ± S.D. * *p* < 0.05, ** *p* < 0.01, *** *p* < 0.001 vs. LPS.

To determine the ability of the peptides to suppress LPS-induced inflammatory responses, human monocyte-derived macrophages were activated with LPS (1 µg/mL) in the presence or absence of peptides (1 mM) for 12 h, and the levels of the pro-inflammatory cytokines IL-6, IL-8 and TNF in the culture supernatants determined by ELISA. Interestingly, with the exception of DIKTNKPVI (p2), all the newly reported peptide fragments significantly decreased the levels of IL-6, IL-8 and TNF in the culture supernatants (Figure 3a–c). Notably, the trend was similar for all three pro-inflammatory cytokines with the anti-inflammatory ability of TNKPVI (p5) comparable to that of the parent decapeptide DIKTNKPVIF (p1) while fragments TNKPVIF (p3) and DIK (p4) had lower anti-inflammatory abilities compared to the parent peptide. While some underlying molecular mechanisms have been elucidated or proposed, there is a lack of information on specific molecular targets of anti-inflammatory food protein-derived peptides, including DIKTNKPVIF. As a preliminary step towards filling this gap, target prediction using SwissTarget showed that human leukocyte antigen (HLA) class I histocompatibility antigen A-3 (P04439) is the most probable inflammation-related protein target in humans for fragments TNKPVIF, DIK and TNKPVI, with 28%, 12% and 39% probability, respectively. DIKTNKPVI and DIKTNKPVIF were not analyzed due to their bulky structures. Antigens are presented to immune cells via HLA class I and class II for immune activation [29]. Future studies will investigate if the potato peptides limit inflammation in autoimmune diseases due to autoreactive T cells via HLA-A3. Furthermore, the removal of Phe from the parent peptide by pepsin, which yielded DIKTNKPVI, led to a complete loss of anti-inflammatory property, with all three cytokine levels similar to those of cells treated with LPS only (Figure 3a–c). Both the parent peptide DIKTNKPVIF and fragment DIKTNKPVI share similar physicochemical properties and ADME/Tox profiles; thus, their structure–function relationship is not clear and may be related to their ability or lack thereof to associate with a specific cell surface or cytosolic molecular targets.

## 4. Conclusions

This study demonstrated that anti-inflammatory DIKTNKPVIF is susceptible to hydrolysis by digestive proteases to generate four fragments, DIKTNKPVI, TNKPVIF, DIK and TNKPVI. Interestingly, the anti-inflammatory activity of DIKTNKPVIF, based on the reduction in the amount of major pro-inflammatory cytokines (IL-6, IL-8 and TNF), in LPS-activated THP-1 cells was retained by fragment TNKPVI. Conversely, fragments TNKPVIF and DIK had moderate anti-inflammatory activities, whereas the removal of only the C-terminal residue of the parent peptide after hydrolysis by pepsin led to a complete loss of bioactivity. Future studies will investigate the molecular targets and signaling pathways mediating the anti-inflammatory effects as well as the structure–function relationship of the potato peptides. Given the low passive gastrointestinal absorption and blood–brain barrier permeability potential of the peptides, future studies will investigate gut-level anti-inflammatory effects where peptide absorption is not required. Taken together, the findings indicate that hexapeptide TNKPVI may be the biostable pharmacophore of the potato patatin-derived anti-inflammatory decapeptide, DIKTNKPVIF.

## Figures and Tables

**Table 1:** Physicochemical properties of the patatin-derived anti-inflammatory decapeptide (p1) and its fragments (p2–p5) released after in silico gastrointestinal digestion.

Peptide or Fragment	MW (Da)	Hydrophobicity Index	Net Charge (pH 1.2)	Net Charge (pH 7)	Boman Index	Instability Index	Aliphatic Index
DIKTNKPVIF (p1)	1174.4	−0.11	2.99	0.76	1.22	20.88	107.0
DIKTNKPVI (p2)	1027.2	−0.43	2.99	0.76	1.68	22.09	118.9
TNKPVIF (p3)	817.98	0.26	2.00	0.76	0.40	37.67	97.14
DIK (p4)	374.43	−0.97	1.99	−0.24	3.12	−21.63	130.0
TNKPVI (p5)	670.81	−0.17	2.00	0.76	0.97	42.28	113.3

MW, experimental molecular weight. Boman index estimates peptide–protein interaction based on solubility properties of amino acid side chains. Instability index estimates the stability of protein in a test tube (value less than 40 indicates that the protein is stable). Aliphatic index estimates the thermostability of globular proteins based on the relative volume occupied by their aliphatic side chains.

**Table 2:** Absorption, distribution, metabolism, excretion and toxicity (ADME/Tox) profile for anti-inflammatory decapeptide and its fragments to predict drug-likeness and suitability for human consumption.

Peptide Sequence	Physicochemical Properties				Toxicity	Lipophilicity		Drug-Likeness		Pharmacokinetics			
	ROTB (*n*) < 10	HBA (*n*) < 10	HBD (*n*) < 5	ESOL Log S	SVM Score (<0.0)	TPSA (Å^2^) < 140	ClogP (o/w) < 5	Bioavailability Score	Lipinski Filter	GIA	P-Gly Substrate	CYP3A4 Inhibitor	BBB Permeability
DIKTNKPVIF	47	18	15	−0.58 (VS)	−1 Non-toxin	469.09	−1.69	0.17	3	Low	Yes	No	No
DIKTNKPVI	42	17	14	0.89 (HS)	−0.83 Non-toxin	439.99	−2.16	0.17	3	Low	Yes	No	No
TNKPVIF	30	12	10	−1.3 (VS)	−0.92 Non-toxin	318.47	−0.93	0.17	3	Low	Yes	No	No
DIK	15	8	6	2.34 (HS)	−0.83 Non-toxin	184.84	−1.4	0.55	1	Low	Yes	No	No
TNKPVI	25	11	9	0.2 (HS)	−0.78 Non-toxin	289.37	−1.58	0.17	3	Low	No	No	No

Abbreviations: ROTB (*n*), rotatable bonds; HBA (*n*), hydrogen bond acceptors; HBD (*n*), hydrogen bond donors; EOSL, estimated solubility [28] with solubility classes in bracket (HS, highly soluble; VS, very soluble); Toxicity SVM score (BIOPEP and ToxinPred), support vector machine score [13]; TPSA (Å^2^), topological polar surface area; CLogP (o/w) consensus logarithm of compound partition coefficient between *n*-octanol and water; Bioavailability score, probability of F > 10% in rat [26]; Lipinski filter (based on Lipinski rules of 5); GIA, gastrointestinal absorption; P-gly substrate, permeability-glycoprotein substrate SVM model (SwissADME); CYP3A4, cytochrome P450 3A4; BBB permeability, blood–brain barrier permeability.

## Data Availability

The article contains data supporting the findings.

## Sample Availability

Not available.
